# Coherent control of radiation patterns of nonlinear multiphoton processes in nanoparticles

**DOI:** 10.1038/srep12040

**Published:** 2015-07-09

**Authors:** Francesco Papoff, Duncan McArthur, Ben Hourahine

**Affiliations:** 1 Department of Physics, SUPA, University of Strathclyde, 107 Rottenrow, Glasgow G4 0NG, UK

## Abstract

We propose a scheme for the coherent control of light waves and currents in metallic nanospheres which applies independently of the nonlinear multiphoton processes at the origin of waves and currents. We derive conditions on the external control field which enable us to change the radiation pattern and suppress radiative losses or to reduce absorption, enabling the particle to behave as a perfect scatterer or as a perfect absorber. The control introduces narrow features in the response of the particles that result in high sensitivity to small variations in the local environment, including subwavelength spatial shifts.

Our ability to enhance light-matter interaction processes in nanophotonics depends on controlling the near and far field optical response of nanostructures. Recently several groups have investigated nonlinear^1^ and linear control based on pulse shaping^2^,^3^, combination of adaptive feedbacks and learning algorithms^4^, as well as optimization of coupling through coherent absorption^5^ and time reversal^6^. In quantum optics, interference between fields was proposed as a way to suppress losses in beam splitters^7^ and has been recently applied to show control of light with light in metamaterials^8^,^9^ and in graphene films^10^. Coherent control of second-harmonic generation using a second pump beam has been recently demonstrated numerically in particles with cylindrical symmetry^11^. For spheres, it was shown in^12^ that the directionality of the emission obtained combining two pump beams results from selection rules that depend on the order of specific process and on the size of the particles.

In this paper, instead, we propose a scheme for the coherent control of scattering and absorption patterns in nanospheres which applies independently of the multiphoton processes at the origin of scattering and absorption, as long as the pump beam is not depleted. The control is extremely sensitive to phase variations and produces a reduction of the absorption and variations in the scattered energy of several orders of magnitude. These features enable applications such as: detection of changes in the position of the particle far smaller than the particle itself, suppression of radiative losses, sensing of variations in the electric permittivity, *ε*, and magnetic permeability, *μ*, and optical switching. With appropriate control beams and pump, one can control the directionality of nonlinearly generated electromagnetic waves not only in a single sphere, but also in a regular array of spheres, for which both the radiation patterns and the spatial positions could be determined. This can be very useful for applications such as optical antennae and for surface enhanced spectroscopy, providing a reference of regularly spaced optical nano beacons for the localization of molecules.

We derive analytically the principles of operation and show that they do not depend on the origin of the response of the particles and are based only on experimentally measurable quantities. The basic idea is to use a control beam coherent with the radiation produced by the nonlinear process: a simple way to realize this is by driving two nonlinear processes of the same order with the same pump, using the output of one of them to control the other, as shown schematically in Fig. 1. As we will see in the following, the identity of the two processes makes the scheme independent of the order of the processes. Furthermore, this approach has the advantage of allowing to control separately internal losses and scattering, enabling the particle to interact with light as a perfect scatterer or as a perfect absorber. For spherical particles this can be done using combinations of electromagnetic waves generated inside and outside the particle, but is impossible using combinations of waves generated only outside the sphere^13^.

We now show how surface and bulk nonlinearity appear in the electromagnetic boundary conditions and use these to find external fields able to control multipolar radiation independently of the specific nonlinear process. We analyze nonlinear processes in which combinations of appropriate order of the pump field and the polarization at frequency *ω*_*p*_ cause bulk and/or surface polarization at frequency *ω* without depleting the pump. In other words, because the nonlinearity is very small, its effect on fields and induced polarization at the frequency of the pump *ω*_*p*_ is negligible and the response at this frequency remains linear. Physically, the nonlinear polarization at frequency *ω* acts as a source of electromagnetic waves at the same frequency which, in turn, excite internal and scattering modes of the particle at frequency *ω* in order to satisfy the boundary conditions. The distribution of the nonlinear polarization within the particle depends only on the pump field at frequency *ω*_*p*_ and it is independent of fields at frequency *ω* that it generates. In this work, the nonlinear polarization plays the same role of the current generated by the power supply in antenna theory (note that for monochromatic fields polarization and current are proportional). For sake of clarity, we stress that there is a difference between induced and nonlinear polarizations. The induced polarization is related to the internal field by the susceptibility and can be eliminated from the Maxwell’s equations that describe the response of the system. On the contrary, the nonlinear polarization is the inhomogeneous term of the Maxwell’s equation that causes the response of the system and cannot be eliminated. This is the same difference that there is between the driving or “external” current and the induced current.

To demonstrate the generality of the control scheme, we consider both particles with linear local responses dominated by divergence free waves (called transversal in the following) and currents confined to the surface, and particles with linear non-local responses^14^,^15^,^16^,^17^,^18^,^19^ that include also irrotational (longitudinal) waves, with internal currents not limited to the surface. In the hydrodynamical model^14^,^15^,^16^,^17^,^18^,^19^, transverse and longitudinal waves are a consequence of representing the free charges in the metal as a fluid with a pressure term of quantum origin that is proportional to the Fermi velocity. In this model the linear interaction of the particle with light is given by the Maxwell equations for the electric and magnetic fields, *E* and *H*, combined with the linearized Navier-Stokes equation for the polarization due to the free current density. It is possible to eliminate polarization and its boundary condition and use only the fields and a boundary condition on the normal component of the displacement, *εE*, which is continuous when there are no charges or polarization layers on the surface^16^,^18^,^19^.

The nonlinear polarization has also been modelled using the hydrodynamical model^17^ or by introducing bulk and surface tensors^20^,^21^. The particular solution of the Maxwell’s equations in the internal medium corresponding to a nonlinear bulk polarization, *P*^*B*^, is *E*^*B*^(*x*) = ∫_*V*_*G*_*E*_(*ω*; *x*, *x*′) ⋅ *P*^*B*^(*x*′)*dx*′, where *G*_*E*_ is the electric dyadic Green’s function at frequency *ω* for the internal medium that, for the hydrodynamical model, includes also longitudinal terms^22^. For the particles most commonly used in experiments, however, nonlocality is important only at the surface^23^ and we can approximate *E*^*B*^(*x*) using the Green’s function without longitudinal terms. We use this Green’s function also when *P*^*B*^ is modelled by the products of tensors with the electric field and its derivatives and the linear response is local^24^. Surface nonlinearities, *P*^*S*^, are instead confined to very thin layers at the surface of the particle and in both models are represented by infinitesimal polarization sheets outside the bulk when the external medium allows interface charge, as in vacuum or air. The surface^25^,^26^ and volume nonlinearities then appear in the boundary conditions at frequency *ω* as


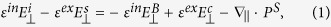



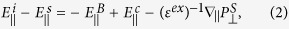



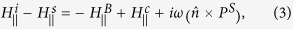


where *i*, *s*, *c* stand for internal, scattered and external control fields, *ex*, *in* for external and internal, and 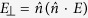, 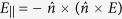 and analogously for the other fields. *E*^*i*^ and *E*^*s*^ are the combination of particles modes (solutions of the homogeneous equations without nonlinear polarizations) that fulfill the boundary conditions. The modes’ amplitudes depend upon the left-hand sides of Eqs (1, 2, 3) which, for any *E*^*B*^, *H*^*B*^ and *P*^*S*^, enable us to find the form of *E*^*c*^, *H*^*c*^ necessary to control the interaction of light with the particle through the amplitudes of the internal and scattering modes, regardless of the nature of the underlining nonlinear processes.

To describe effects that are most easily observed experimentally, we concentrate here on the control of two modes and outline later how the theory generalizes to an arbitrary number of modes. As a consequence of the rotational invariance, the only modes that are spatially correlated at the surface of a sphere are internal and scattering electric or magnetic multipoles with the same value of *l* (total angular momentum) and *m* (angular momentum along the direction of propagation of the pump). Electric (magnetic) multipoles have magnetic (electric) fields with null radial component^27^. We note that there are another two types of multipolar waves for the external medium that are relevant to this work: incoming, which propagate inward and have a divergence at the center, and regular, which are used to expand waves with amplitudes bounded everywhere, as the plane waves. All types of electric or magnetic multipoles with the same indexes *l* and *m* have the same angular dependence in spherical coordinates^27^, but different radial dependence. In our notation


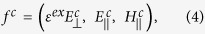



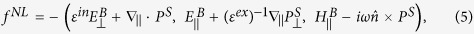


are the surface vector functions of the control field (*f*^*c*^) and of the nonlinear (*NL*) sources that appear in the boundary conditions, Eqs (1, 2, 3), for a pump of amplitude *a*^*p*^ = 1 in arbitrary units. The real amplitude and phase of *f*^*c*^ are encoded in the complex amplitude *a*^*c*^. For any pair of internal and scattering modes, *i*_*lm*_, *s*_*lm*_, for which we adopt the same notation as for *f*^*c*^, the amplitudes 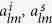 are given by


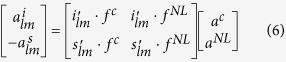


where the scalar product indicates the sum of the overlap integrals (i.e. the spatial correlations) of all the components with *a*^*NL*^ = (*a*^*p*^)^*N*^ the amplitude of *f*^*NL*^ and *N* the order of the nonlinear process. Note that *s*_*lm*_, *i*_*lm*_, are either transverse electric or transverse magnetic, but for ease of notation we do not specify which type they are. The biorthogonal mode^28^ 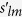 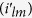 is orthogonal to all modes other than *s*_*lm*_ (*i*_*lm*_). For spheres (and for any finite set of modes) the biorthogonal modes can be found analytically and depend on all internal and scattering modes with the same *l* and *m*, correlated at the surface of the sphere, according to the formula provided in the Methods section.

To clarify the meaning of Eq. (6), we note that for *f*^*NL*^ = 0, i.e. in the absence of nonlinear processes, and *f*^*c*^ ≠ 0, Eq. (6) is a mathematically compact and efficient formulation of the Mie theory, in which the source of internal and scattering modes is the incident field at the surface of the sphere, *f*^*c*^. The amplitudes of the modes are determined by the boundary conditions and the material properties at frequency *ω* are fully included in the structure of the modes. When *f*^*c*^ = 0 and *f*^*NL*^ ≠ 0, the nonlinear polarizations *P*^*B*^ and *P*^*S*^ act as external sources and excite internal and scattering modes: Eq. (6) gives us the amplitudes of these modes from the boundary conditions at frequency *ω*, generalizing an approach pioneered in^29^ for Raman and fluorescence processes due to molecules embedded in dielectric particles. When *f*^*c*^ and *f*^*NL*^ are both non null and coherent, Eq. (6) gives the amplitudes of the modes, which in this case are originated by the interference of *f*^*c*^ and *f*^*NL*^ at the surface of the particle.

We can find any value for the ratio of the mode amplitudes, 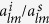 — i.e. we can vary the response of the particle on the controlled modes from that of a perfect scatterer with 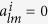, 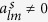 to that of a perfect absorber with 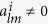, 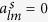 — by changing the amplitudes of control and pump fields as long as the matrix in Eq. (6) is invertible, i.e. as long as the condition 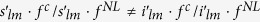 is fulfilled. For any pair of modes, *s*_*lm*_ and *i*_*lm*_, this condition is satisfied only if 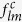 and 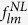, which are the terms with the same angular dependence of *s*_*lm*_ and *i*_*lm*_ in the expansions of *f*^*c*^ and *f*^*NL*^, are different. This is the case for any nonlinear process and a regular control beam generated outside the particle by a laser, because 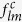 is a regular multipole containing radial functions of argument *k*^*e*^*r*, while 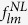 is a combination of an outgoing multipole with radial functions of argument *k*^*i*^*r* (the bulk term) and a product of *N* — the order of the nonlinear process — regular multipoles with radial functions of argument 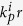 (the surface term), where *k*^*e*^ (*k*^*i*^) is the wavenumber of the external (internal) medium at frequency *ω* and 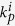 is the wavenumber of the internal medium at frequency *ω*_*p*_. Note that it is not possible to replace *f*^*NL*^ with a beam generated outside the particle by another laser because such external beams can always be expanded in terms of the same regular multipolar spherical waves^13^ as *f*^*c*^, which means that the matrix in Eq. (6) is not invertible.

We remark that while the sphere can behave as a perfect scatterer or a perfect absorber, the mechanism described here is significantly different from coherent perfect absorption^5^. This is a resonant process which can be understood as the time reversal of lasing and happens for particular values of the dissipation, when there are interference patterns inside the system able to trap a specific incoming mode indefinitely. We show instead that any spherical multipolar wave can be either trapped inside or expelled from the particle by the interference pattern formed by the appropriate combination of an incident wave with the surface polarization and the internal wave induced by the nonlinear process. This happens for any value of the dissipation, but for particular values of the amplitude of the control wave. To understand this effect from a physical point of view, let us recall that the Huygens-Fresnel principle, formally proved in the Stratton-Chu theorem^30^, states that the secondary waves emitted from any closed surface by a scattered field propagating outward from any point inside the surface vanish anywhere inside the surface and add constructively outside to reconstruct the scattered field. Note that the tangent components of the field at the surface of the particle are the source of the secondary waves, acting as free surface currents. If we use an internal field instead of a scattering field, the secondary waves cancel outside the surface and add constructively inside. We can then understand perfect scattering or perfect absorption in terms of the formation of equivalent surface currents, which are combination of physical surface currents proportional to surface polarizations and tangent incident field components, which can radiate only either outside or inside the surface. Here the equivalent surface currents are those used in the boundary conditions of Eqs (2,3): the terms proportional to the nonlinear surface polarization and the tangent components at the surface of the sphere of the control field *E*^*c*^, *H*^*c*^, incident to the surface from the outside, and of the field *E*^*B*^, *H*^*B*^, incident to the surface from the inside. Another advantage of this form of coherent control is that narrow resonant features can be observed in the response of the controlled particle when the material parameters or the frequency are changed while the control amplitudes are kept constant, as a result of the dependence of the modes on the parameters that are changed. The values of frequency or material parameters at which these narrow features reach minima or maxima can be changed by changing the control amplitudes. This allows us to engineer particle responses with high sensitivity to change in external parameters, as we show in the following.

Ideally, the control beam should leave the amplitudes of all the other modes unchanged, which means that the control beam should have the same angular dependence on the surface of the sphere as the controlled multipole. For this reason, multipolar waves are the ideal control beams for single sphere applications: in principle these waves could be produced by using an appropriate distribution of electric and magnetic dipoles over a closed surface surrounding the sphere. In practice this is impossible and it is very difficult to realize good approximations of multipolar spherical waves centered on the particle to be controlled. Here we consider mainly control beams consisting of plane waves, which can be more easily and precisely implemented over small volumes, and use polarization and angle of incidence to optimize the coupling with the multipoles. This procedure can be performed algorithmically using the expansion of plane waves in regular multipoles^27^, whose coefficients depend on the polarization and the angle of incidence. Dipolar terms with *l* = 1 are dominant in the plane wave expansions at the surface of the sphere; however, it is possible to control a multipole with *l* > 1 without affecting dipole terms simply by combining two plane waves non-collinear with the pump to form a control field that is invariant under a rotation of *π* around the direction of propagation of the pump. This procedure can be generalized to remove from the control field multipole terms of order lower than a given value using similar symmetry arguments. Therefore plane waves can be used very effectively to control the radiation patterns by fixing the amplitudes of the dominant modes. Furthermore, using pump and control beams that are approximately plane waves allows one to have the same control condition at regularly spaced locations, which can be applied to the control of arrays of spheres placed at the intersection of the equiphase planes of the pump and the control beams, as long as the spheres are sufficiently far apart that their mutual interaction is negligible. In this case, the angles of incidence are discrete and the number of control beams affects both the number of modes controlled and the geometry of the array.

We point out that the theory we described explains the principles of the coherent control that we propose, but no *a priori* theoretical knowledge is necessary to implement such control. From an operational point of view, it is only necessary to determine experimentally the multipolar fields excited by the nonlinear sources; once these are known, the control field *E*^*c*^, *H*^*c*^ is chosen considering the multipolar terms one wants to control and the desired mode amplitudes are found by adjusting the amplitude and phase of the control field. Schematics of the proposed set-up are shown in Fig. 1. The control wave is sent along one of the directions in which the multipolar wave to be controlled is maximal, and a detector collects light coming from a solid angle centered on another direction of maximal field, without receiving light directly from the control beam. The optimal values of amplitude and phase of the control beam are determined by modulating the phase periodically and adjusting the amplitude so that the detected signal shows the largest variation. This procedure is fully self-consistent and requires only information provided by the experiment itself. To demonstrate numerically the properties discussed above, we apply this control technique to a gold nanosphere of 50 nm radius using plane waves for the pump and the control beam: we keep the pump constant, vary amplitude and phase of the control and compare local and nonlocal responses for second harmonic generation. For particles of this size, bulk nonlinearities are negligible^20^,^24^ and the nonlinear polarization sheet is dominated by the radial component which excites an electric dipole with *l* = 1, *m* = 0 and electric quadrupoles with *l* = 2, *m* = 0 and *l* = 2, *m* = ±2. Both hydrodynamical and surface tensor models for nonlinear polarizations have been tested and give qualitatively similar results but with some quantitative differences. We have verified our control theory on all models. For the following calculations we assume a local response with nonlinear polarization 
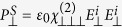
, where the second-order susceptibility tensor component 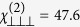 in units of 3.27 × 10^−17^ m/V^20^. The amplitudes of the multipoles generated by the nonlinear polarization and of the control beams are of the same order and scale linearly with 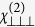. In Fig. 2 we control the internal and scattering modes *i*_10_ and *s*_10_ of the electric dipole. In Fig. 2a the amplitude of the control beam is chosen so that the amplitude of *s*_10_, 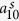, can vanish at the appropriate phase; we show the intensity of the field scattered in a direction orthogonal to both pump and control: other multipoles do not emit in this direction so the intensity has the same dependence of 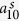 and show an extremely sharp variation. The light scattered in a solid angle centered on this direction can be monitored in an experiment to optimize the control beam; note that the phase sensitivity shown in Fig. 2a allows us to map the position of the particle with a resolution Δ*λ*/*λ = *ΔΦ/2*π*, where *λ* and Φ are the wavelength and relative phase of the control beam respectively. This provides a deeply sub-wavelength spatial resolution when no other multipole radiate in the solid angle of detection and the sensitivity of the detector allows one to monitor the logarithm of the signal. The optimal solid angle can be found by considering the known radiation patterns of the multipoles^27^. The ratio of the amplitudes 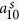 and 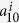 shows that we find the condition for a perfect scatterer in Fig. 2a and for a perfect absorber in Fig. 2b, while the amplitudes of the other modes (not shown) are not affected by the control beam. By removing the dominant internal mode, we can minimize the total absorption, which is very useful to reduce heating and, as a consequence, increase stability in experiments. Figure 3 shows the radiation patterns with and without control in the equatorial plane *θ* = 90° of the sphere. In Fig. 4a, we control the intensity of the field scattered in a direction at *π*/2 with respect to the control beam and at *π*/4 with respect to the pump by changing the amplitudes of the modes of the electric quadrupole with *l* = 2, *m* = ±2, as shown in Fig. 4b. Even in this case we can observe a subwavelength variation of the intensity. In Fig. 5a we use an incoming multipolar wave with *l* = 2, *m* = 2 to control the scattering for the same sphere and in the same direction as in Fig. 4a. Note that in this case the variation of the intensity is smaller than in Fig. 4a because the multipolar control wave affects only the *l* = 2, *m* = 2 mode, as can be seen from Fig. 5b. We need two control beams to control independently the modes *l* = 2, *m* = 2 and *l* = 2, *m* = −2 in order to improve on the result shown in Fig. 4a, but Fig. 5a shows that using incoming multipolar waves is not necessarily more effective than using plane waves. Finally, in Fig. 6 we show how the sensitivity to phase variation can be applied to monitor small variations in the dielectric permittivity of the host medium; similar results could be achieved with variations of the magnetic permeability. With the intensity and phase of the pump and control beams optimised to suppress the *s*_10_ mode for a particular environment, *ε*^*ex*^, (corresponding to Δ*ε*^*ex*^ = 0 in Fig. 6) we observe a strong sensitivity to small changes in *ε*^*ex*^ in the scattered intensity. As the modes of the system depend upon the local environment, the relative phase and amplitude of the control beam required to maintain suppression of the modes change with it. When we vary the optimised amplitude of the control field by ±20% we observe in Fig. 6a that the curve of the scattered intensity drifts, so that the minima no longer occurs at Δ*ε*^*ex*^ = 0, and the sensitivity decreases slightly. In Fig. 6b we observe that the sharpness of the feature in the scattering intensity reduces significantly when the relative phase of the control beam, Φ^*c*^, is changed from the optimised value, but the position of the minima in this case does not drift. Generalizing this approach to include any number of modes and external incident waves is straightforward and explained in the Methods section.

In conclusions, we have presented a scheme for the coherent control of light and currents in nanospheres, identifying extreme sensitivity to variations of the relative phase of two coherent incident waves that can lead to novel applications. The theory we have presented can be easily generalized to particles with other shapes: in most cases the Mie approach based on separation of variables does not apply, but the principal mode theory can be used^31^. The control of a larger number *N* of modes is in principle straightforward and requires to adjust the amplitudes of the pump and of *N* − 1 control beams. From a practical point of view, spatial light modulators and configurable array mirrors could provide an effective way to implement control schemes for several modes, providing an efficient and adaptable coupling to a high number of modes with a low number of beams, each with a complex profile made up by the superposition of several plane waves. Finally, we have been concerned here with the control of radiation patterns resulting from multiphoton processes, but the theory we developed can also be used to control absorption and scattering of external fields through appropriate distributions of surface polarization.

## Methods

The biorthogonal modes are given by the formula


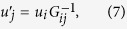


where *u*_1_ = *s*_*lm*_, *u*_2_ = *i*_*lm*_, *G*^−1^ is the inverse of the (Gram) matrix with elements *G*_*ij*_ = (*u*_*i*_ ⋅ *u*_*j*_) and we sum over repeated indexes. When longitudinal modes are present^15^, we can include them simply by defining *u*_3_ as the longitudinal mode spatially correlated to *s*_*lm*_ and *i*_*lm*_.

Generalizing Eq. (6) to include any number of modes and external incident waves is straightforward, as the amplitude of each mode requires only the scalar product of its biorthogonal mode with the sum of all the fields incident on the surface and the surface polarization. For any set of incident electromagnetic waves, 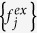, the first column of the matrix in Eq. (6) is replaced by two matrices: the matrix *S* with elements 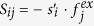 and the matrix *I* for the internal modes with elements 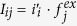, where *i* = (*l*, *m*). When *f*^*NL*^ = 0, the amplitudes of the modes are given by the product of these two matrices with the amplitudes of the incident waves. When *f*^*NL*^ ≠ 0, the amplitudes of the modes are given by the product of the augmented matrices 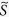 and 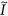, with 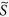 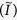 obtained by adding to *S* (*I*) the column 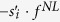 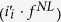, with a column vector containing the amplitudes of the incident waves and of *f*^*NL*^. Control of the amplitudes of *N* modes can be achieved with *N* − 1 control beams when *f*^*NL*^ ≠ 0 and the matrix 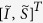 is invertible.

## Additional Information

**How to cite this article**: Papoff, F. *et al.* Coherent control of radiation patterns of nonlinear multiphoton processes in nanoparticles. *Sci. Rep.* **5**, 12040; doi: 10.1038/srep12040 (2015).

## Figures and Tables

**Figure 1:**
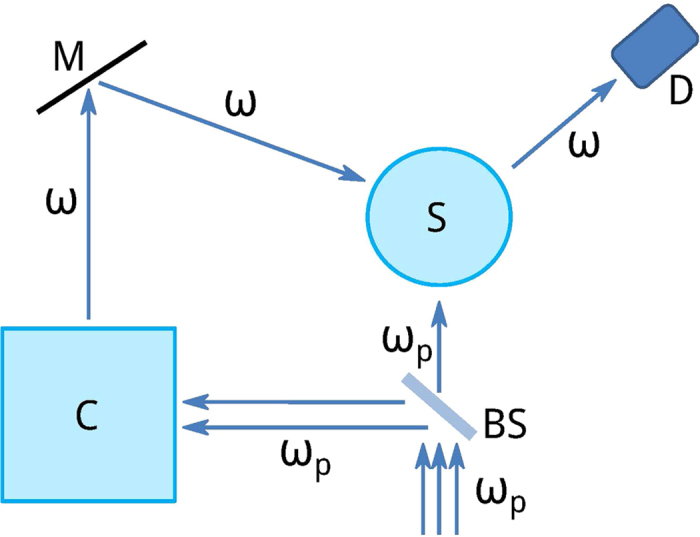
Schematic set-up. Set up (not to scale) for the implementation of the control scheme proposed in the main text. The pump beam at frequency *ω*_*p*_ is divided by a beam splitter (BS) into two beams that drive the same nonlinear process. Light at frequency *ω* is collected from the control generator (C) and directed to the nanosphere (S) along a direction where the controlled multipole has a maximum. The process is monitored by a detector (D) that collects light from a solid angle centered on a direction of maximal multipolar emission with no direct illumination from the control beam. The three parameters to be changed to control the output from the sphere are direction of incidence, power and relative phase of the control beam.

**Figure 2:**
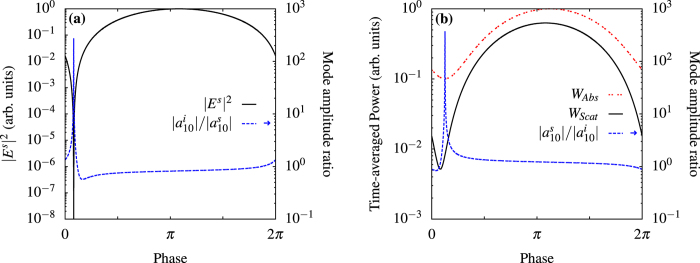
Control of the dominant dipole mode. We use gold spheres of radius 50 nm and a Lorentz-Drude model to calculate the dielectric function of gold^32^. Scattered field intensity along a direction orthogonal to both pump and control beams and mode amplitudes against relative phase of control beam. In this figure and in the following ones, we used a pump amplitude *a*^*p*^ = 1 in arbitrary units with *ω*_*p*_ = 281.76 THz. In all plots, arrows in the key indicate when values should be read from the secondary axis. (**a**) Control at *π*/2 with respect to the pump, on *l* = 1, *m* = 0 internal and scattering modes, at frequency *ω* = 563.52 THz with control beam amplitude |*a*^*c*^| = 1.47 × 10^−12^ in arbitrary units to find perfect absorption on the controlled modes. The point where 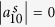, within the numerical resolution, has been removed to avoid compressing the plot of the coefficients’ ratio for all other values of the phase. (**b**) Total absorbed and scattered time-averaged powers versus the relative phase of the control beam. |*a*^*c*^| = 1.28 × 10^−12^ to find perfect scattering on the controlled mode. The total absorption, which includes also non controlled modes, has a minimum exactly where the dominant internal mode is suppressed (perfect scattering on the controlled mode). For a phase of the control beam close to the value corresponding to suppression of the internal mode there is a minimization of the amplitude of the *l* = 1, *m* = 0 scattering mode which is clearly observable in the total scattered power. As in (**a**) the point of perfect scattering has been removed.

**Figure 3:**
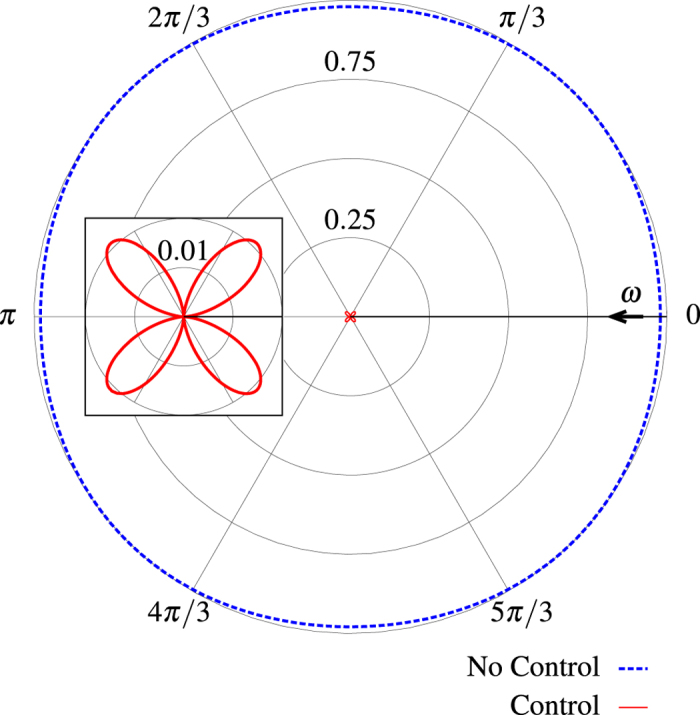
Radiation patterns with and without control. Radiation pattern along the plane *θ* = 90° for the same particle, frequency, and control as Fig. 2a. Inset shows an enlargement of the *s*_22_ quadrupole, the arrow indicates the direction of the control beam.

**Figure 4:**
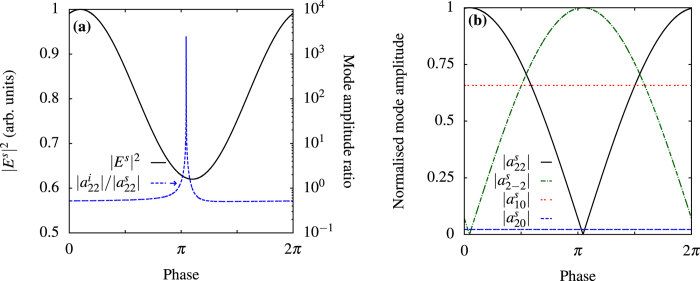
Control of quadrupolar modes with a plane wave. Same particle and frequency as Fig. 2, with point of perfect absorption removed from plots. (**a**) Control at *π*/4 with respect to the pump, on *l* = 2, *m* = ±2 internal and scattering modes, |*a*^*c*^| = 1.32 × 10^−11^ to find perfect absorption on the *l* = 2, *m* = 2 modes. (**b**) Amplitudes on the modes excited, showing that the control beam affects only the modes *l* = 2, *m* = ±2.

**Figure 5:**
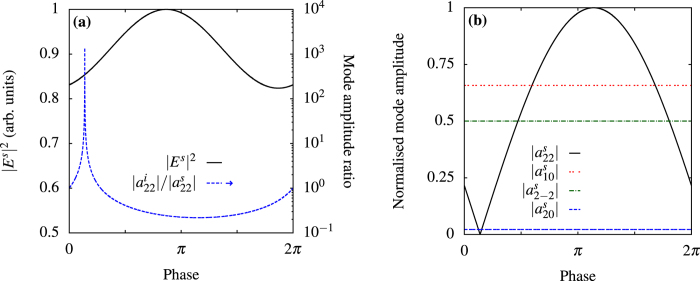
Control of quadrupolar modes with an incoming spherical wave. Same particle and frequency as Fig. 2, with point of perfect absorption removed from plots. (**a**) Same scattering direction as in Fig. 4a, but using as the control beam an incoming multipole with *l* = 2, *m* = 2 that affects only the amplitude of the modes with *l* = 2, *m* = 2, as shown in (**b**) The smaller variation in the scattered intensity is due to the fact that this control beam has no effect on the mode *l* = 2, *m* = −2.

**Figure 6:**
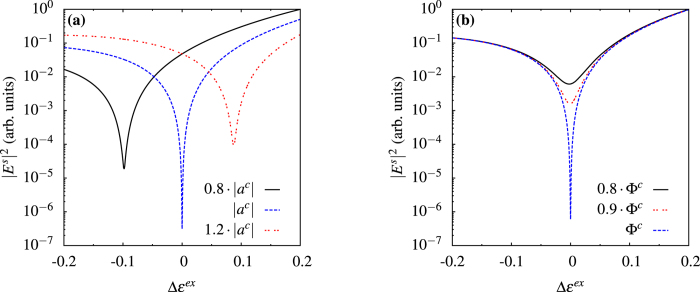
Sensitivity to local environment. Same particle and frequency as Fig. 2. The intensity and phase of the pump and control beam are optimised to suppress the *s*_10_ mode at Δ*ε*^*ex*^ = 0, as in Fig. 2a. The directional scattered intensity is extremely sensitive to small changes in the external dielectric permittivity (blue-dashed curve). This effect enables the measurement of the local environment. (**a**) When |*a*^*c*^| is changed by ±20% and the relative phase is kept constant, as the control is no longer optimised at Δ*ε*^*ex*^ = 0, the curve of the scattered intensity drifts (black-solid/red-dotted curves) but we still observe sharp features. (**b**) The sharpness of the feature in the scattering intensity reduces significantly when the relative phase of the control beam, Φ^*c*^, is changed from the optimised value by 10% (red-dotted curve) or 20% (black-solid curve) while |*a*^*c*^| is kept constant.
